# The prognostic value of Dickkopf-3 (Dkk3), TGFB1 and ECM-1 in prostate cancer

**DOI:** 10.3389/fmolb.2024.1351888

**Published:** 2024-05-24

**Authors:** Zainab Al Shareef, Mahmood Y. Hachim, Amal Bouzid, Iman M. Talaat, Natheer Al-Rawi, Rifat Hamoudi, Ibrahim Y. Hachim

**Affiliations:** ^1^ Research Institute of Medical and Health Sciences, University of Sharjah, Sharjah, United Arab Emirates; ^2^ College of Medicine, University of Sharjah, Sharjah, United Arab Emirates; ^3^ College of Medicine, Mohammed Bin Rashid University of Medicine and Health Sciences, Dubai, United Arab Emirates; ^4^ Faculty of Medicine, Alexandria University, Alexandria, Egypt; ^5^ College of Dental Medicine, University of Sharjah, Sharjah, United Arab Emirates; ^6^ Division of Surgery and Interventional Science, University College London, London, United Kingdom

**Keywords:** prostate cancer, Dickkopf-3, TGF-β signalling pathway, ECM-1, prognosis

## Abstract

Prostate cancer (PCa) is considered one of the most common cancers worldwide. Despite advances in patient diagnosis, management, and risk stratification, 10%–20% of patients progress to castration-resistant disease. Our previous report highlighted a protective role of Dickkopf-3 (DKK3) in PCa stroma. This role was proposed to be mediated through opposing extracellular matrix protein 1 (ECM-1) and TGF-β signalling activity. However, a detailed analysis of the prognostic value of DKK3, ECM-1 and members of the TGF-β signalling pathway in PCa was not thoroughly investigated. In this study, we explored the prognostic value of DKK3, ECM-1 and TGFB1 using a bioinformatical approach through analysis of large publicly available datasets from The Cancer Genome Atlas Program (TGCA) and Pan-Cancer Atlas databases. Our results showed a significant gradual loss of *DKK3* expression with PCa progression (*p* < 0.0001) associated with increased DNA methylation in its promoter region (*p* < 1.63E-12). In contrast, patients with metastatic lesions showed significantly higher levels of *TGFB1* expression compared to primary tumours (*p* < 0.00001). Our results also showed a marginal association between more advanced tumour stage presented as positive lymph node involvement and low *DKK3* mRNA expression (*p* = 0.082). However, while *ECM1* showed no association with tumour stage (*p* = 0.773), high *TGFB1* expression showed a significant association with more advanced stage presented as advanced T3 stage compared to patients with low *TGFB1* mRNA expression (*p* < 0.001). Interestingly, while *ECM1* showed no significant association with patient outcome, patients with high *DKK3* mRNA expression showed a significant association with favourable outcomes presented as prolonged disease-specific (*p* = 0.0266), progression-free survival (*p* = 0.047) and disease-free (*p* = 0.05). In contrast, high *TGFB1* mRNA expression showed a significant association with poor patient outcomes presented as shortened progression-free (*p* = 0.00032) and disease-free survival (*p* = 0.0433). Moreover, *DKK3, TGFB1* and *ECM1* have acted as immune-associated genes in the PCa tumour microenvironment. In conclusion, our findings showed a distinct prognostic value for this three-gene signature in PCa. While both DKK3 and TGFB1 showed a potential role as a clinical marker for PCa stratification, ECM1 showed no significant association with the majority of clinicopathological parameters, which reduce its clinical significance as a reliable prognostic marker.

## Introduction

Prostate cancer (PCa) is still one of the most common male cancers (Greenberg et al., 2013) and one of the top five leading causes of death (Rawla, 2019; Barsouk et al., 2020). Moreover, many reports have highlighted an increase in the number of PCa cases diagnosed annually with the prediction that those numbers might increase in the near future (Mistry et al., 2011). An important element in determining the optimal management plan for patients with PCa is risk stratification (Greenberg et al., 2013; Yamazaki et al., 2021). Gleason score, PSA levels and clinical stage are considered important elements in risk stratification and prediction of tumour progression as well as recurrence (Yamazaki et al., 2021). The Gleason score is one of the main components of PCa prognosis and a risk stratification tool and is mainly based on the assessment of architectural features and glandular de-differentiation (Chen and Zhou, 2016; Tagai et al., 2019; van Leenders et al., 2020).

PCa patients may develop resistance to initial hormone therapy (10%–20%) and progress to a more advanced stage known as a castration-resistant disease (Vellky and Ricke, 2020; Harris et al., 2009; Yamazaki et al., 2021). This presents a clinical challenge, and there is a need to discover new prognostic biomarkers that can predict tumour progression and recurrence accurately (Frantzi et al., 2020).

Many reports highlighted the important role of cancer cell interactions with the reactive stroma in determining tumour behaviour, spread and progression. Some factors in the tumour microenvironment have tumour-inhibitory effects that might improve patient prognosis (Baghban et al., 2020), despite the stromal compartment in many cancers being pro-tumourigenic (Lopes-Coelho et al., 2018; Valkenburg et al., 2018). Recently, we revealed a protective role of stromal Dickkopf-3 (DKK3) in PCa (Al Shareef et al., 2018). Moreover, we also observed an inverse correlation between DKK3 and transforming growth Factor Beta Induced 1 (TGFB1) expression and that the activity of DKK3 was affected in different ways by TGFB1 and ECM-1 (Al Shareef et al., 2018; Niehrs, 2006; Kikuchi et al., 2021).

DKK3 is a member of the DKK family, secreted glycoproteins that negatively regulate Wnt signalling, although in the case of DKK3, this must be through a different mechanism (Veeck and Dahl, 2012). This might be attributed to the distinct amino acid sequence of DKK3, compared to other DKK family members, in the region of the LRP6-binding site (Niehrs, 2006; Kikuchi et al., 2021). Reports suggest that the tumour suppressive activity of DKK3 is related to its negative regulation of the β-catenin activity (Lee et al., 2020).

Previous reports highlighted a reduction in DKK3 expression in aggressive human cancer cells including basal breast cancer, melanoma, and hepatocellular carcinoma (HCC) through the promotor hypermethylation (Veeck and Dahl, 2012). Moreover, ectopic overexpression of DKK3 in some cancer cell lines inhibits their proliferation or induces apoptosis. Together, these studies suggest DKK3 has a tumour-suppressive function in various cancers (Hsieh et al., 2004; Kuphal et al., 2006; Lorsy et al., 2016). In contrast, some other reports highlight a tumour-promoting role of DKK3, for example, in oesophageal adenocarcinoma and oral squamous cell carcinoma (SCC), where it increases tumour cell proliferation and migration (Katase et al., 2013; Wang et al., 2015).

In the current study, we performed a comprehensive analysis of the prognostic value of *DKK3, TGFB1* and *ECM-1* as a multiple-gene prognostic signature in PCa using bioinformatics approaches.

## Materials and methods

### Gene expression evaluation in PCa nad normal samples

The publicly available application TNM plot (https://tnmplot.com/analysis/) was used to evaluate the mRNA expression levels of the genes of interest in normal (*n* = 106), tumour (*n* = 283) and metastatic (*n* = 6) samples (Bartha and Gyorffy, 2021). For statistical significance, the Kruskal–Wallis test was used to compare the gene expression levels among normal, tumour and metastatic tissue with *p* < 0.01 used as a cut-off value for statistical significance. In addition, using cBioPortal (cbioportal.org), we retrieved and analysed mRNA data (RNA SeqV2) of 493 patients with prostate adenocarcinoma from the Cancer Genome Atlas (TGCA) and Pan-Cancer Atlas. Log-rank test *p*-values were used to evaluate the statistical significance between the low and the high expression level (classified according to above and below mean mRNA expression of each biomarker. The Chi-squared test was used to evaluate the association between each biomarker and clinicopathological parameters including tumour stage as well as patient outcome. Moreover, the DNA methylation profiles retrieved from the TCGA database were assessed in order to evaluate the *DKK3* promoter DNA methylation levels using the UALCAN portal. DNA methylation analysis was carried out using normalized beta values from 502 PCa patients and 50 healthy controls considering beta value cut-offs of hyper-methylation [beta value: 0.7–0.5] or hypo-methylation [beta-value: 0.3–0.25].

### Evaluation of the prognostic capacity

To evaluate the prognostic capacity of each marker of interest, we used the CANCERTOOL database, (http://web.bioinformatics.cicbiogune.es/CANCERTOOL), which is a comprehensive portal that aims to investigate different genes and their association with clinical data including disease progression, pathologic, and molecular characteristics, to evaluate the expression levels of our markers in normal *versus* malignant tissues.

### Correlation analysis of gene expression and immune infiltration in PCa tumour microenvironment

The relationship between *DKK3, TGFB1* and *ECM1* and immune cell infiltration in the in PCa microenvironment was explored using the Tumour IMmune Estimation Resource (TIMER) (https://cistrome.shinyapps.io/timer/), which consists of deconvolution and comprehensive analysis of tumour-infiltrating immune cells in various types of cancer. The association between *DKK3, TGFB1* and *ECM1* expression and the abundances of immune infiltrates including B cells, CD4^+^ T cells, CD8^+^ T cells, neutrophils, macrophages, and dendritic cells in the PCa tumour microenvironment was assessed, using Spearman’s test (*p* < 0.05).

### Construction of gene-gene interaction network and functional enrichment analysis

To assess the functional association between the studied genes *DKK3, TGFB1* and *ECM1* and their related connected genes, the gene-gene interaction network was constructed based on a large data of functional-related data sets including genetic and protein interactions, physical interaction, co-expression, co-localization, and common pathway, using GeneMANIA bioinformatics tool with defaults parameters (Warde-Farley et al., 2010). Genes can be linked by the interacted network based on variable attributes. The network includes nodes that represent genes while edges represent connections. Besides, functional enrichment analysis on Gene Ontology terms including biological process (BP), cellular component (CC), and molecular function (MF) categories was conducted using the Enrichr database (Xie et al., 2021) to explore the biological significance and common pathways between the *DKK3, TGFB1* and *ECM1* genes. The hypergeometric distribution cut-off for the functional enrichment analyses was a *p*-value of <0.05.

## Results

### DKK3 is downregulated in PCa samples compared to normal tissue

**FIGURE 1:**
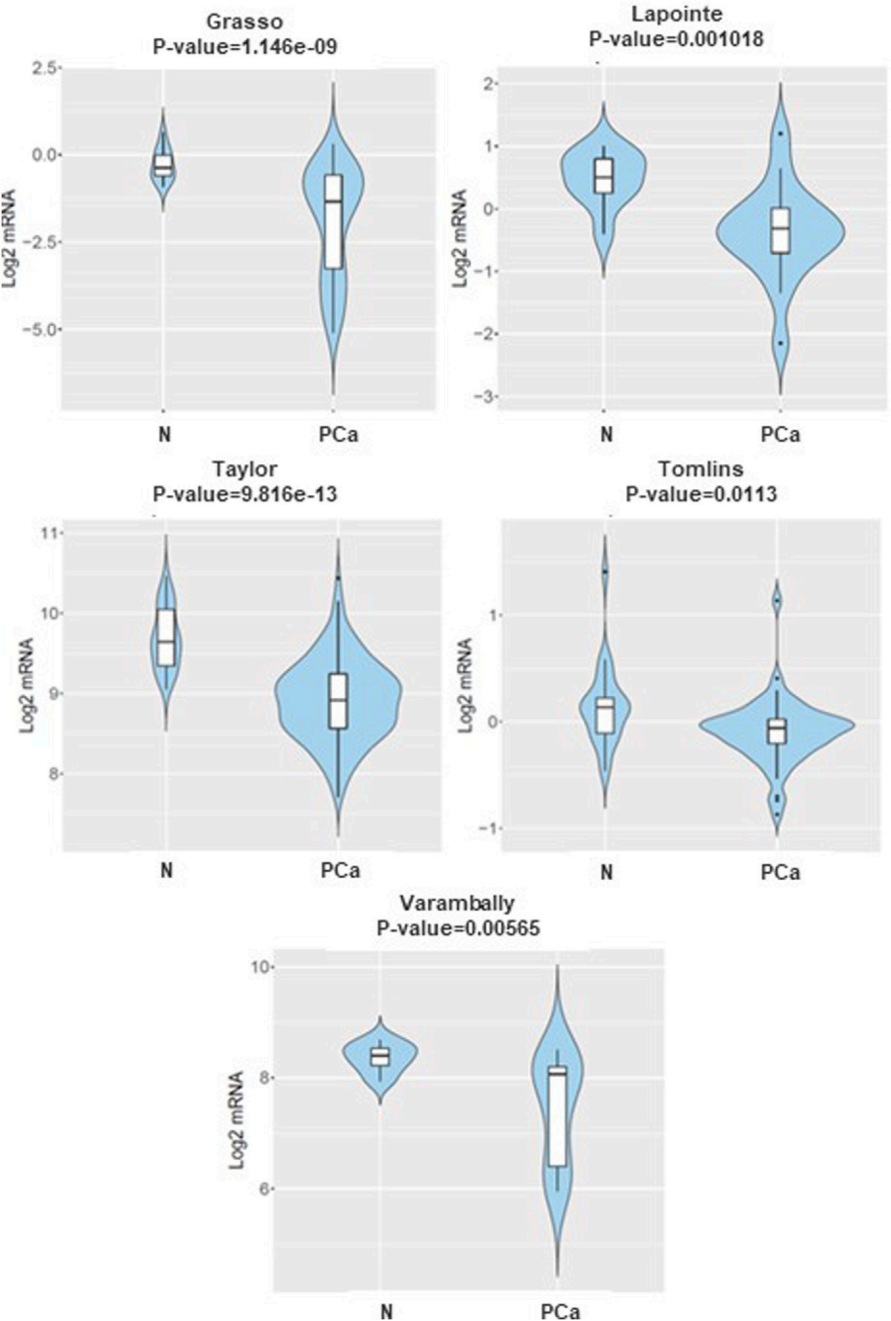
The mRNA expression of DKK3 in normal versus prostate cancer samples CANCERTOOL database. Violin plots show the expression of DKK3 in non-tumoral tissue (N) and primary tumours (PT) in various datasets of the CANCERTOOL database.

To improve our understanding of the role of *DKK3, TGFB1* and *ECM-1* genes in tumourigenesis, we initially investigated the expression levels of *DKK3* in PCa samples compared to their normal counterparts using the CANCERTOOL database. Different datasets were used including Glinksy et al., Grasso et al., Lapointe et al., Taylor et al., TCGA (RNA-seq), Tomlins et al., and Varambally et al. (Figure 1). Our results showed a significant lower level of *DKK3* in PCa samples compared to samples obtained from their normal counterparts. Similarly, *TGFB1* showed also significant downregulation in PCa samples compared to normal counterparts. In contrast, ECM-1 levels showed no significant differences between the normal and PCa samples (Supplementary Figure S1).

**FIGURE 2:**
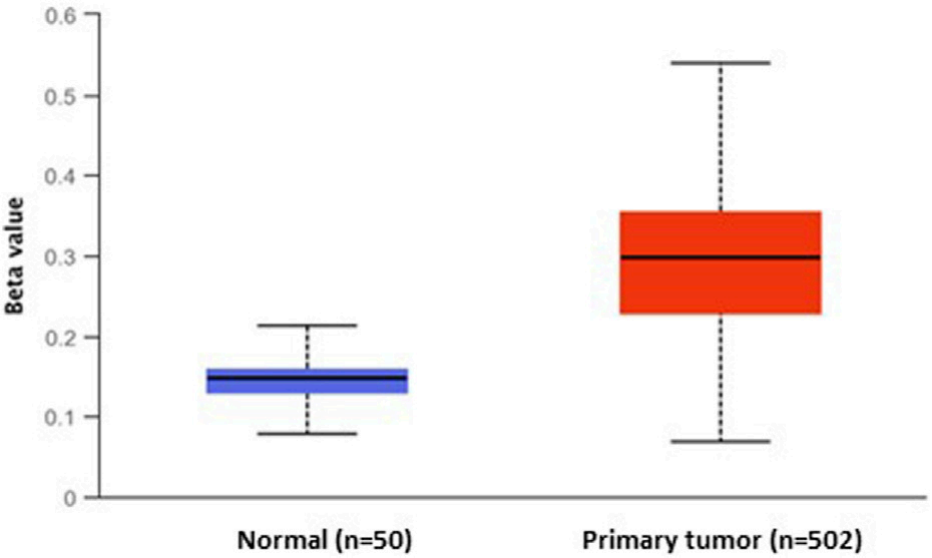
The differential DNA methylation levels of DKK3 promoter between normal and primary prostate tumours.

Moreover, the evaluation of DNA methylation levels in the promoter region of *DKK3* using TCGA publicly available methylome data showed significant DNA methylation differences between primary PCa and normal samples (*p* < 1.62 E-12). Indeed, the *DKK3* promoter was highly hypermethylated among PCa cases (n = 502), while it was hypomethylated in the normal samples (n = 50). Our findings indicate that there is a notable decrease in the expression of DKK3 in PCa samples from various datasets, indicating that the promoter region of the DKK3 gene may be affected by DNA methylation, leading to its silencing (Figure 2).

### The value of DKK3 expression as a marker of tumour progression in PCa samples

**FIGURE 3:**
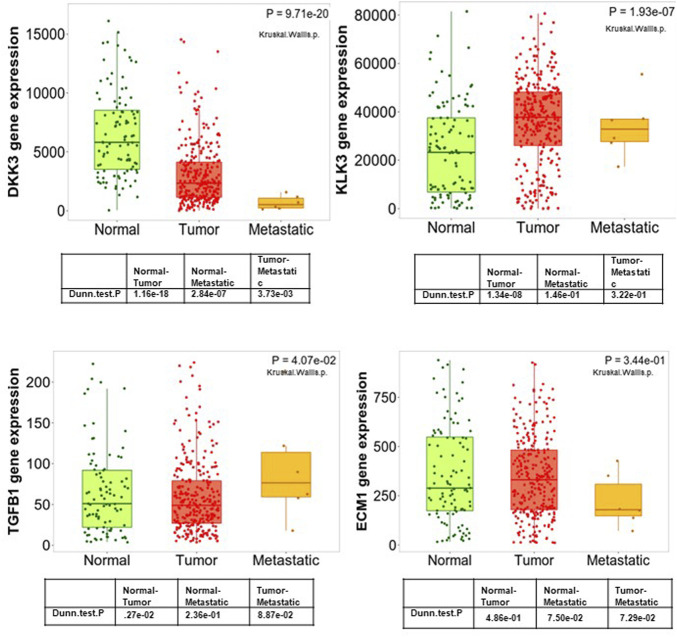
Boxplots of the mRNA expression of DKK3, ECM1, TGFB1 and PSA (KLK3) in a large patient cohort using the TNM plot database.

To improve our understanding of the role of *DKK3, ECM-1* and *TGF-β* in PCa tumourigenesis, we evaluated their expression in samples representing different stages of PCa progression (Figure 3). To achieve this, we analysed *DKK3, ECM-1* and *TGFB1* expression in normal prostate, primary tumours and samples obtained from more advanced diseases presented as patients with tumour metastases. Interestingly, our results showed a gradual and significant downregulation of *DKK3* expression during tumour progression. Indeed, the expression levels of *DKK3* showed significant downregulation in tumour samples compared to normal. Further downregulation was observed between tumour samples and samples obtained from patients with metastatic tumours (*p* < 0.0001). In contrast, while *TGFB1* expression was comparable between normal and tumour samples, metastatic lesions showed a significantly higher level of *TGFB1*, compared to both primary tumour and normal samples (*p* = 0.04). Moreover, while *ECM1* expression levels were higher in tumour samples compared to normal tissue, *ECM1* expression was significantly lower in metastatic samples compared to tumour, as well as normal samples (*p* < 0.0001). As a reference, we also investigated the expression levels of the classical marker *PSA* (*KLK3*) in the same samples. Our findings showed that while PSA levels initially increased in the tumour samples compared to normal, those levels were significantly lower in metastatic samples, compared to tumours (*p* < 0.0001). In summary, our results highlight the possible benefits of *DKK3* expression as a marker of PCa progression, owing to its gradual loss during tumourigenesis.

### The association between *DKK3, ECM-1 and TGFB1* mRNA expression and tumour staging and grade in a large patient cohort

**FIGURE 4:**
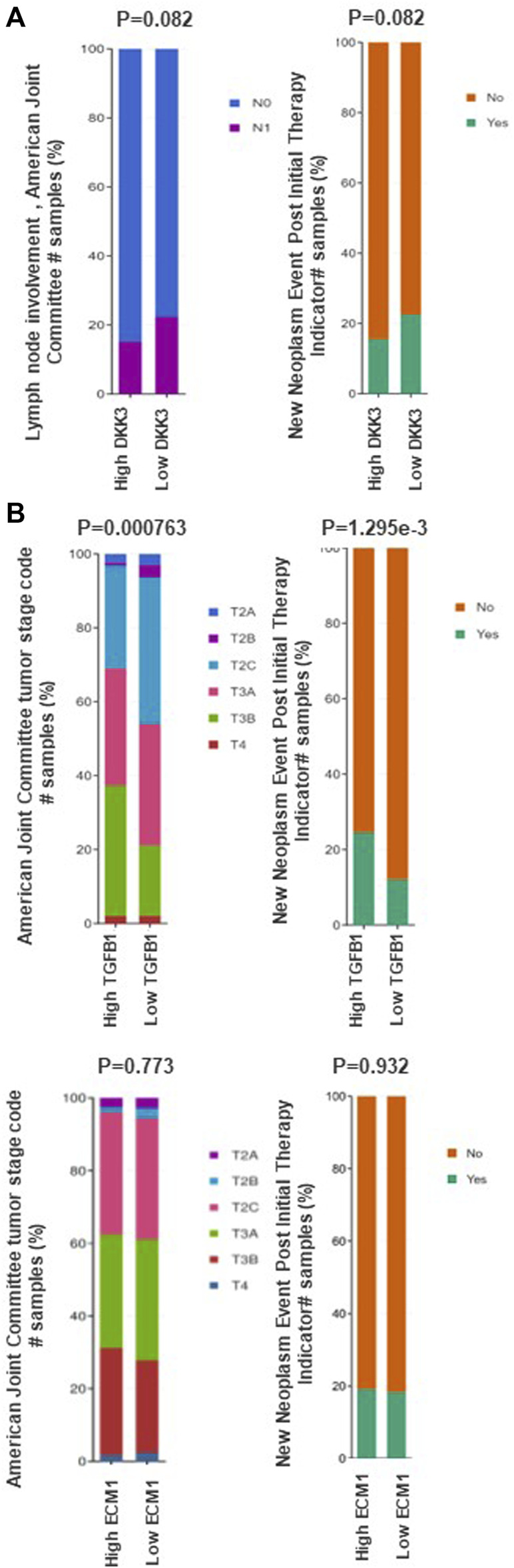
The association between DKK3, ECM1 and TGFB1 mRNA expression and clinicopathological features of prostate cancer. **(A)** The association between DKK3 and prostate cancer clinicopathological characteristics using data from prostate adenocarcinoma (TCGA) through cBioPortal tool. **(B)** The association between ECM1 and TGFB1 mRNA expression prostate cancer clinicopathological characteristics using data from prostate adenocarcinoma (TCGA) through cBioPortal tool.

Next, we evaluated the mRNA expression of the three genes in association with the tumour stage using the prostate adenocarcinoma (TCGA, PanCancer Atlas) cohort (Figure 4). Our results showed a marginal significance (*p* = 0.082) between *DKK3* mRNA expression and LN involvement (Figure 4A). Patients with higher *DKK3* mRNA expression showed less chance of positive lymph node involvement (N1) compared to patients with lower *DKK3* mRNA expression. In comparison, while *ECM1* showed no association with tumour stage (*p* = 0.773), *TGFB1* showed a significant association with tumour stage (Figure 4B). Patients with high *TGFB1* mRNA expression presented with the more advanced T3A (tumour spread outside the prostate, but not invading seminal vesicles) and T3B (tumour spread outside prostate with seminal vesicles invasion) stages, compared to patients with low *TGFB1* mRNA expression (*p* < 0.001).

Interestingly, while patients with high *DKK3* mRNA expression showed marginally significant lower chance of having recurrence presented as new neoplastic events post initial therapy (*p* = 0.082), Patients with higher *TGFB1* mRNA expression showed a significant higher chance of recurrence presented as new neoplastic events post initial therapy compared with patients with low *TGFB1* mRNA expression (*p* < 0.001). In contrast, *ECM1* mRNA expression showed no significant association with new neoplastic events post initial therapy (*p* = 0.932) (Figure 4).

The analysis between the mRNA expression of the three markers with tumor grade showed that while both DKK3 and ECM1 showed no significant variation in samples from different Gleason grade, TGFB1 mRNA expression was significantly higher in patients presented with higher gleason score (G8,9&10) in the TCGA dataset (*p* = 0.0001) (Supplementary Figure S2).

### The correlation between DKK3 mRNA expression and both TGFB1 and ECM1 in prostate cancer samples

Further analysis was performed to investigate the correlation between DKK3 expression and TGFB1 and ECM1 in prostate cancer samples. Indeed, this might better demonstrate the interplay between DKK3 and these markers. While our analysis showed no significant correlation between ECM1 and DKK3 mRNA levels (R = 0.07, *p* = 0.23), our results showed a significant and negative correlation between DKK3 mRNA expression levels and TGFB1 (R = -0.17, *p* < 0.0001). Additional analysis was performed to investigate the association between DKK3 and KLK3, which is most relevant marker for PCa diagnosis and management, and its expression is regulated by the upstream markers, such as AR. Our analysis showed no significant correlation between DKK3 mRNA expression levels and KLK3 expression (R = -0.09, *p* = 0.14) (Supplementary Figure S3).

### The association between DKK3, ECM1 and TGFB1 and patient outcome

**FIGURE 5:**
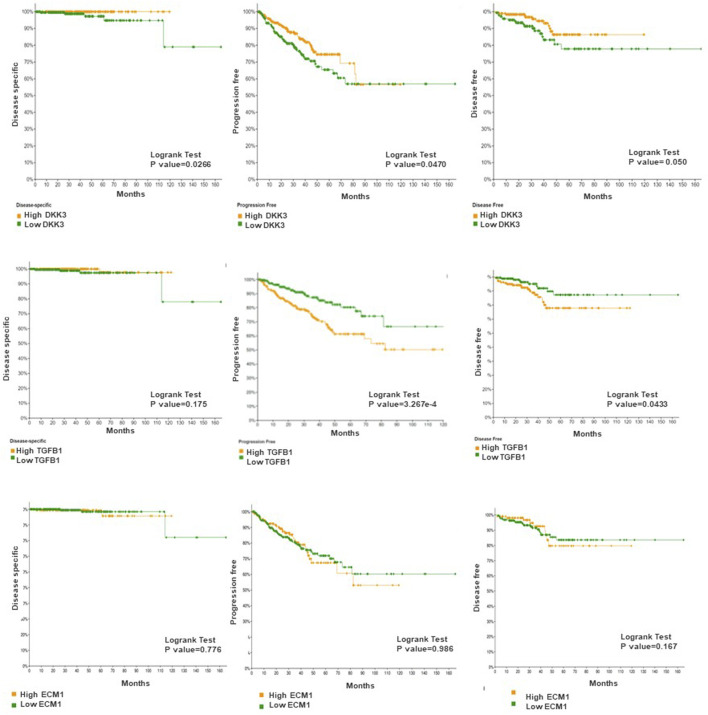
The association between DKK3, ECM1 and TGFB1 mRNA expression and patient survival. Log-rank test p-values were used to evaluate the statistical significance between the low and the high expression level.

Next, we evaluated the association between our markers and patient outcome presented as progression-free survival (Figure 5). While ECM-1 showed no significant association with patient outcome progression-free survival (*p* = 0.986), disease specific (*p* = 0.776) and disease free survival (*p* = 0.167). Both DKK3 and TGFB1 showed a significant and contrasting association with patient outcomes. While patients with high DKK3 mRNA expression showed a significant association with favourable outcomes presented as prolonged progression-free survival (*p* = 0.0470), disease specific (*p* = 0.0266) and disease free survival (*p* = 0.050), high TGFB1 mRNA expression showed a significant association with poor patient outcome presented as shortened progression-free survival (*p* = 3.267e-4) and disease free survival (*p* = 0.0433).

### *DKK3, TGFB1* and *ECM1* act as the immune-associated genes in PCa

**FIGURE 6:**
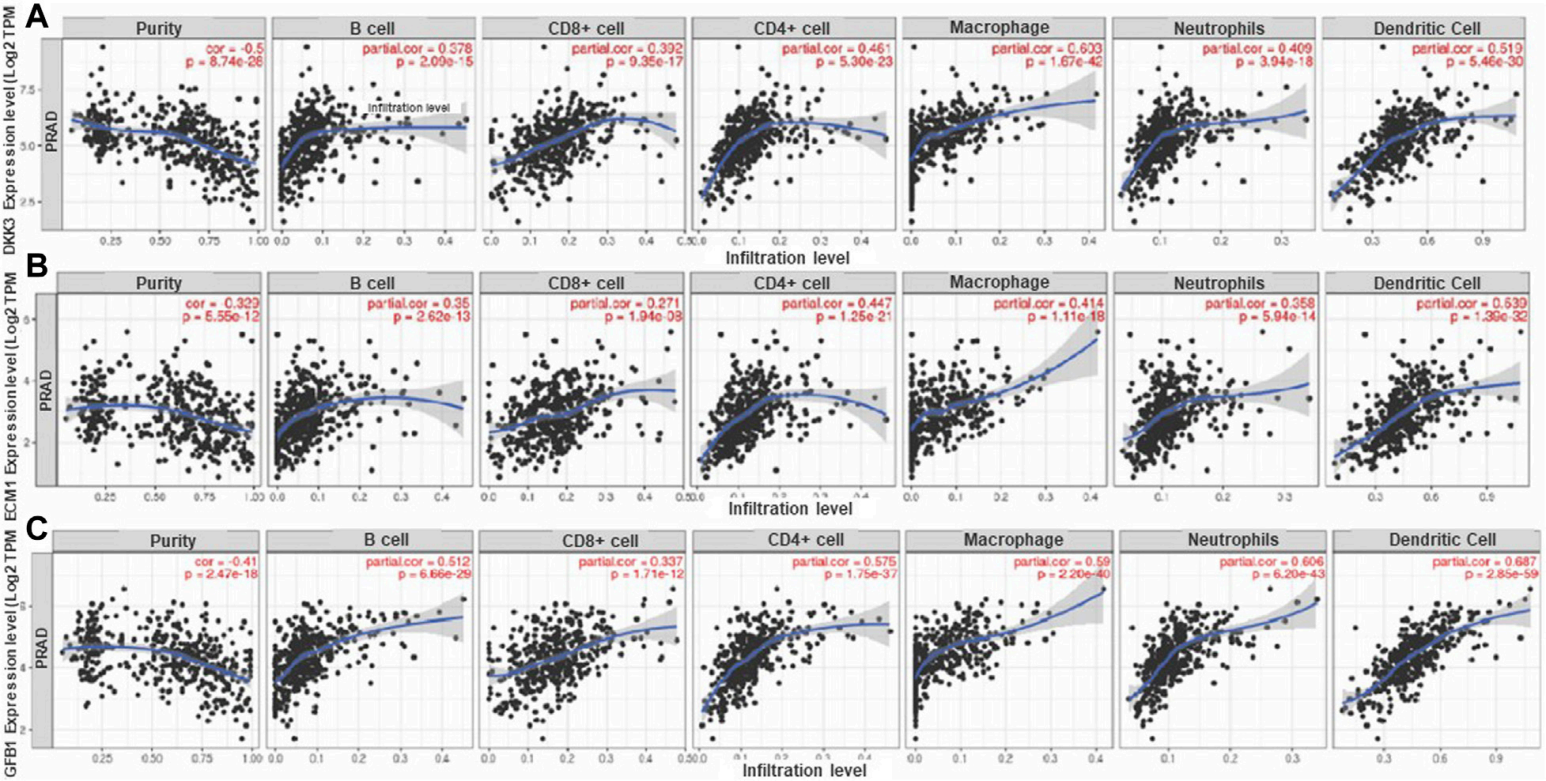
Correlation of **(A)** *DKK3*, **(B)** *ECM1* and **(C)** *TGFB1* gene expression level with immune cell infiltration in PCa. The scatterplots displayed Spearman's rho value correlation and statistical significance showing the potential interplay between *DKK3, TGFBI* and *ECMI* biomarkers and immune cells (B-Cell, CD8+ T Cell, CD4+ T Cell, Macrophage, Neutrophil and Dendritic Cell) in the prostate cancer tumor microenvironment. The log2 TPM gene expression values are presented on the y-axis, the average immune cell infiltration levels are presented on the x-axis. The blue curve and gray area in the figures show the general trend direction. TPM: transcripts per million. (Data generated from TIMER Webtool).

We were also interested to analyze the relationship between *DKK3, TGFB1* and *ECM*1 and immune cell infiltration in prostate cancer (Figure 6). The results showed that gene expression level against tumor purity is highly significant for the three biomarkers with negative Spearman’s rho values (−0.5, −0.329 and −0.41), respectively in *DKK3, TGFB1* and *ECM1* suggesting that these biomarkers are highly expressed in the microenvironment of PCa (Figure 6). Moreover, all biomarkers have a positive significant correlation with B cells, CD4^+^ T cells, CD8^+^ T cells, Neutrophils, Macrophages, and Dendritic cells. Importantly, *DKK3* has the highest correlation with macrophage at 60%, while *ECM1* showed the highest correlation at about 54% with dendritic cells and *TGFB1* has the highest correlation with neutrophil and dendritic cells at 60% and 68% respectively. Importantly, all biomarkers had a better correlation with CD4 than CD8.

### Biological functions and interconnections of the *DKK3, TGFB1 and ECM1* genes

**FIGURE 7:**
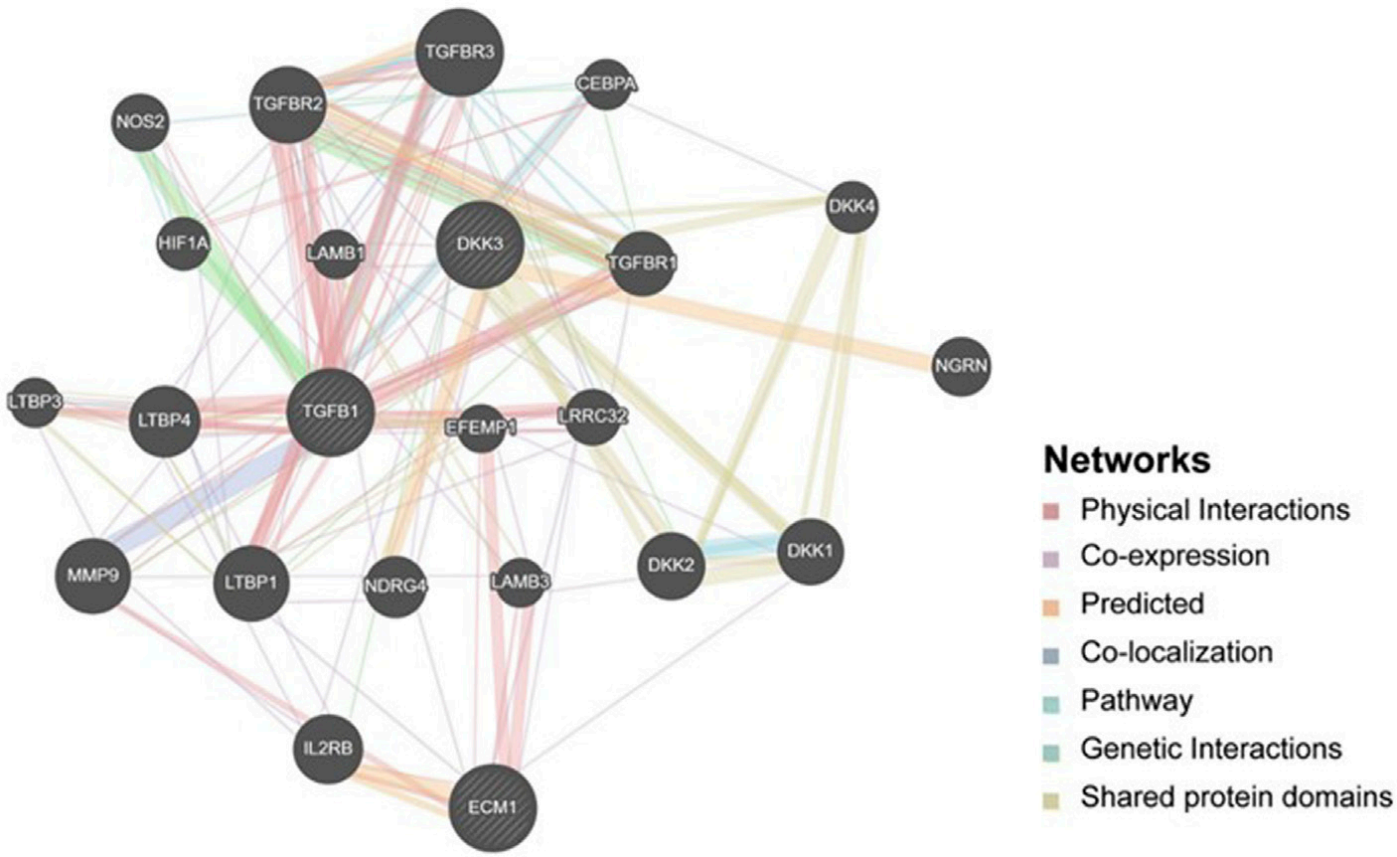
Gene-Gene interaction network including *DKK3*, *TGFB1* and *ECM1*. It shows interaction strength (edge thickness), interaction type (colour), multiple edges between nodes, and protein score (node size) defined using a stylesheet constructed with GeneMANIA. The interconnections between studied genes were evaluated based on physical interaction, co-expression, predicted, co-localization, common pathway, genetic interaction and shared protein domains.

To explore the potential mechanisms that involve *DKK3, TGFB1 and ECM1* in the carcinogenesis of PCa, we explored GeneMania to build up a gene–gene interaction network for these biomarkers. As shown in Figure 7, the network revealed that *DKK3, TGFB1* and *ECM1* share many genes within the same gene family or similar protein domain under a series of cooperators suggesting that there is a possibility of the interplay between the 3 markers through interaction with proteins from a similar family. For instance, *MMP9* presents a key marker in the network that showed physical interconnection with *TGFB1* and *ECM1*, and co-expression with *DKK3* suggesting the potential direct/indirect connections between these particular biomarkers. Knowing that *MMP9* codes for a matrix metalloproteinase protein that is reported to promote metastasis and angiogenesis through decomposition of the extracellular matrix in several tumours which further supports the possible involvement of *DKK3, TGFB1* and *ECM1* in the PCa progression.

**FIGURE 8:**
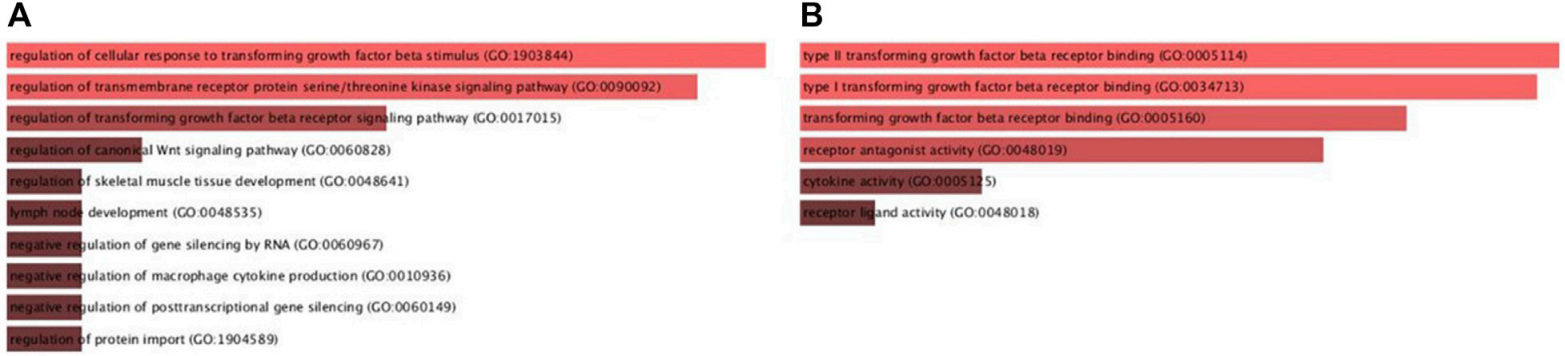
Gene ontologies enrichment analysis **(A)** Biological Process and **(B)** Molecular Functions of *DKK3, TGFBI* and *ECMI* using Enricher database.

Furthermore, gene ontologies including biological process and molecular function were performed to reveal the functional enrichment of *DKK3, TGFB1* and *ECM1* and their co-operators. The significant annotated pathways were represented in Figure 7. Interestingly, it identifies cytokine activity (GO:0005125, *p*-value = 0.02) and macrophage-related pathways such as negative regulation of macrophage cytokine production (GO:0010936, *p*-value = 7.5E-04) among the differentially regulated pathways involving mainly *TGFB1* as a key player which further supports the findings of immune cell infiltration in PCa (Figure 8). Additionally, the regulation of canonical Wnt signaling pathway (GO:0060828, *p*-value = 4.74E-04) was identified among the relevant significantly regulated biological processes involving *TGFB1* and *DKK3,* which could be of a pivotal role in PCa pathogenesis, proliferation and resistance to treatment. Taken together, these findings highlight the potential utility of *DKK3, TGFB1 and ECM1*as prognostic markers for PCa.

## Discussion

Previous reports highlighted the role of the Dickkopf (Dkk) gene family in determining cell fate during embryonic development (Hoang et al., 2004; Krupnik et al., 1999; Hoang et al., 2004; Niehrs, 2006). Moreover, the *DKK3* gene was found to encode essential intracellular regulators of cellular proliferation (Leonard et al., 2017). A tumour suppressor role of DKK3 was suggested since ectopic expression of Dkk3 was found to reduce cancer growth, in contrast, loss of Dkk3 expression induces hyperproliferation of cells (Veeck and Dahl, 2012; Fujii et al., 2014). In addition, other reports also found lower levels of DKK3 in various tumours including gastric, ovarian, lung, bladder and PCa (Kurose et al., 2004; Chim et al., 2008; Yue et al., 2008; Ueno et al., 2011).

Our earlier report proposed a tumour suppressor role of *DKK3* in PCa through opposing *TGFBI* and *ECM-1* function (Al Shareef et al., 2018). However, a comprehensive analysis of the prognostic role of *DKK3*, as well as its relationship with the expression of *TGFB1* and *ECM1* in a large sample size of PCa was not investigated. Here we have analysed the prognostic value of *DKK3, TGFB1* and *ECM1* and their association with well-known prognostic clinicopathological parameters in PCa using multiple cohorts in various publicly available databases. Additionally, we conducted an investigation into the pathways involved in the interaction between DKK3, TGFB1, and ECM1.

This study reveals that *ECM1* and *TGFB1* are upregulated in cancerous tissues in comparison to normal tissues. Conversely, *DKK3* expression was found to be downregulated in malignant tissue in comparison to normal tissue. These results align with prior research that has demonstrated decreased expression of *DKK3* in various types of cancer as compared to healthy tissue (Kurose et al., 2004; Chim et al., 2008; Yue et al., 2008; Ueno et al., 2011). This also supports our previous notion that DKK3 might exert a protective role against PCa (Al Shareef et al., 2018) and highlighted a possible role of its loss in PCa tumourigenesis.

Our results also showed a gradual loss of DKK3 mRNA expression between and during cancer progression with its least expression in samples obtained from patients with metastasis. Interestingly, our results showed that DKK3 expression was more able to predict tumour progression than KLK3 (coding for PSA). Indeed, PSA is considered one of the most relevant markers for PCa diagnosis and management (Bonk et al., 2019). Moreover, recent reports highlighted KLK3 as a possible predictive marker for molecular lymph-node staging in PCa patients (Lunger et al., 2021). However, our analysis showed that while KLK3 expression was significantly upregulated in primary cancer samples compared to normal, its expression was not significantly variable between metastatic and primary tumour samples. Indeed, this statement suggests that the clinical usefulness of PSA (KLK3) in assessing tumor progression is greatly restricted, and instead emphasizes the potential benefits of utilizing DKK3 expression as an indicator of cancer progression. In addition, our results also showed a significant association between low DKK3 expression and poor patient outcomes represented as shortened disease-specific, disease free and progression-free survival. While the association between DKK3 expression and patient outcome in PCa was not thoroughly investigated, recent reports showed a significant association between low DKK3 expression and reduced recurrence-free survival in breast cancer, cervical cancer, colorectal cancer, endometrial cancer and gastric cancer (Wang et al., 2012; Xu et al., 2012; Ryu et al., 2013; Lorsy et al., 2016).

Our results also highlighted a possible interplay between DKK3, TGFB1 and ECM1 in PCa through modulation of some biological processes and molecular functions including members of TGF-β signaling and the Wnt signaling pathway. Wnt Signaling was found to play an essential role in the modulation of PCa microenvironment to promote drug resistance as well as cancer stem cell expansion and self-renewal (Kypta and Waxman, 2012). Our analysis also identified pathways involved in the regulation of macrophage cytokine production as one of the enriched pathways in the regulation of DKK3, TGFB1 and ECM1 function in PCa. This highlighted a possible mechanism through which those genes modulate PCa microenviroment through regulation of macrophage function, which is now believed to play a role in PCa progression and metastatic cascades of PCa (Lo and Lynch, 2018). Further studies are still needed for comprehensive analysis for the role of DKK3, TGFB1 and ECM1 in PCa including the use of human single cell/spatial analysis of human Pca. Indeed this might improve our understanding to the prognostic role and their role in pathogenesis of prostate including progression and metastasis.

## Conclusion

To summarize, our study utilized *in silico* analyses to investigate the potential utility of *DKK3*, *TGFB1* and *ECM1* as prognostic markers for PCa. Our findings suggest that while *DKK3*, *TGFB1* could serve as promising biomarkers for predicting disease progression and patient outcomes, *ECM1* expression showed no significant association with various clinicopathological parameters. For that reason, only *DKK3* and *TGFB1* can be used as a reliable prognostic markers in prostate cancer and the he incorporation of these biomarkers into clinical practice could enhance PCa patient stratification and facilitate the adoption of tailored therapeutic strategies for each individual. Furthermore, our analysis identified a group of key pathways that appear to be central in the interplay between DKK3, TGFB1 and ECM1, resulting in the modulation of the tumour microenvironment.

## Data Availability

The original contributions presented in the study are included in the article/Supplementary Material, further inquiries can be directed to the corresponding author.
