# Supplementary figures and images for: The prognostic value of Dickkopf-3 (Dkk3), TGFB1 and ECM-1 in prostate cancer
Source: Front Mol Biosci. 2024 May 24;11:1351888. doi: 10.3389/fmolb.2024.1351888 (PMC11157039; doi:10.3389/fmolb.2024.1351888)

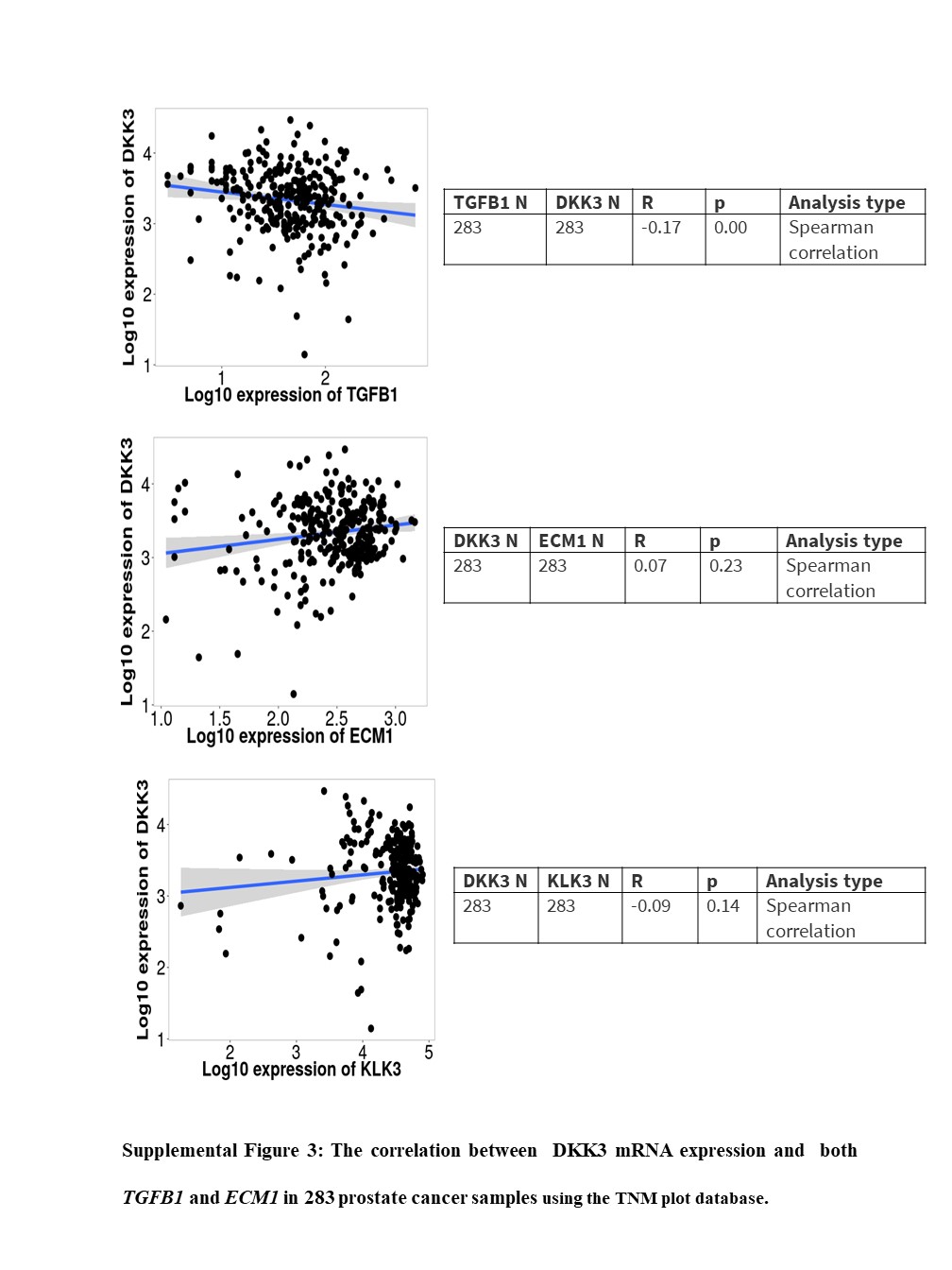

Supplement: Supplementary file 1 [file Image3.JPEG]

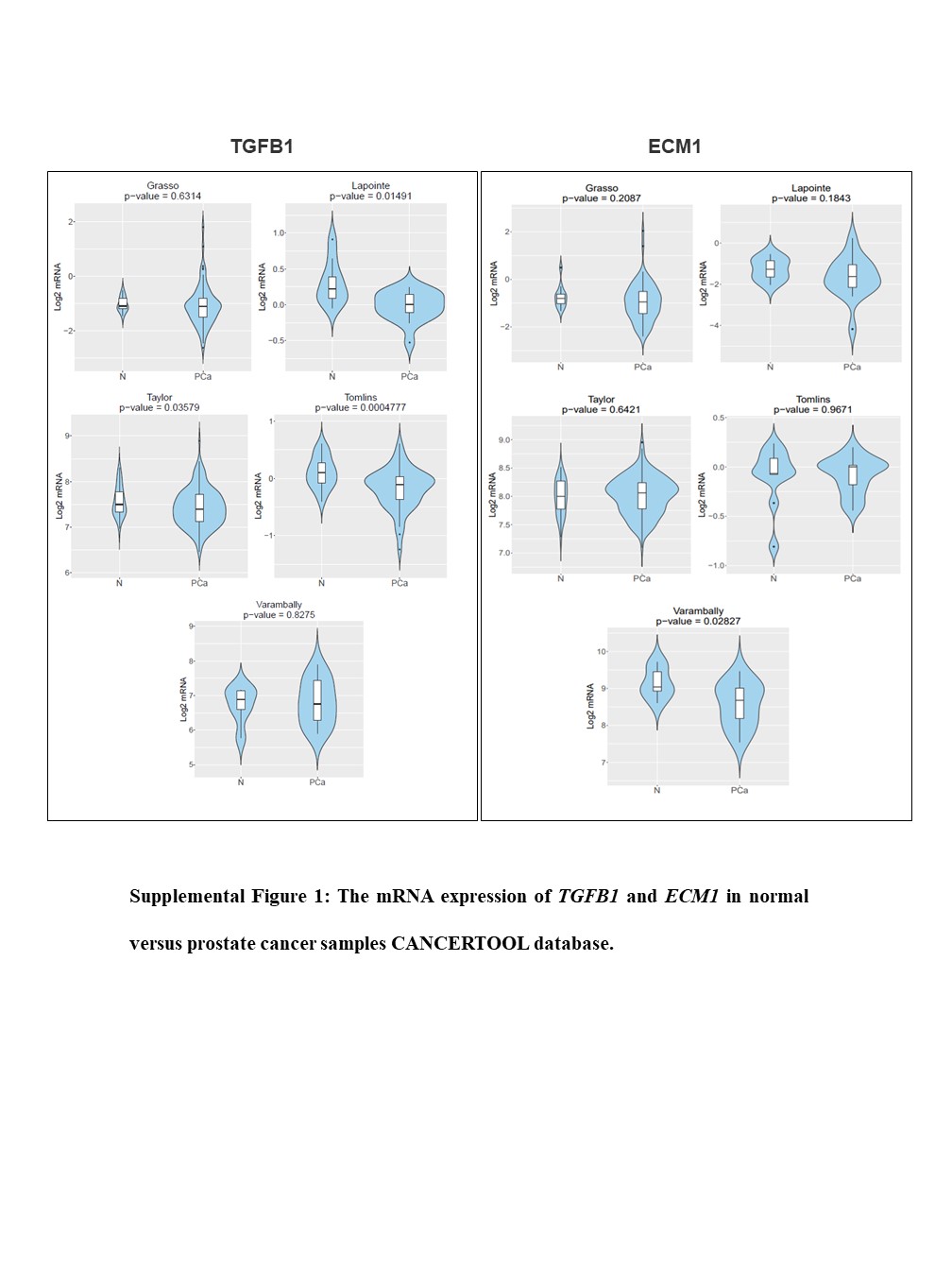

Supplement: Supplementary file 2 [file Image1.JPEG]

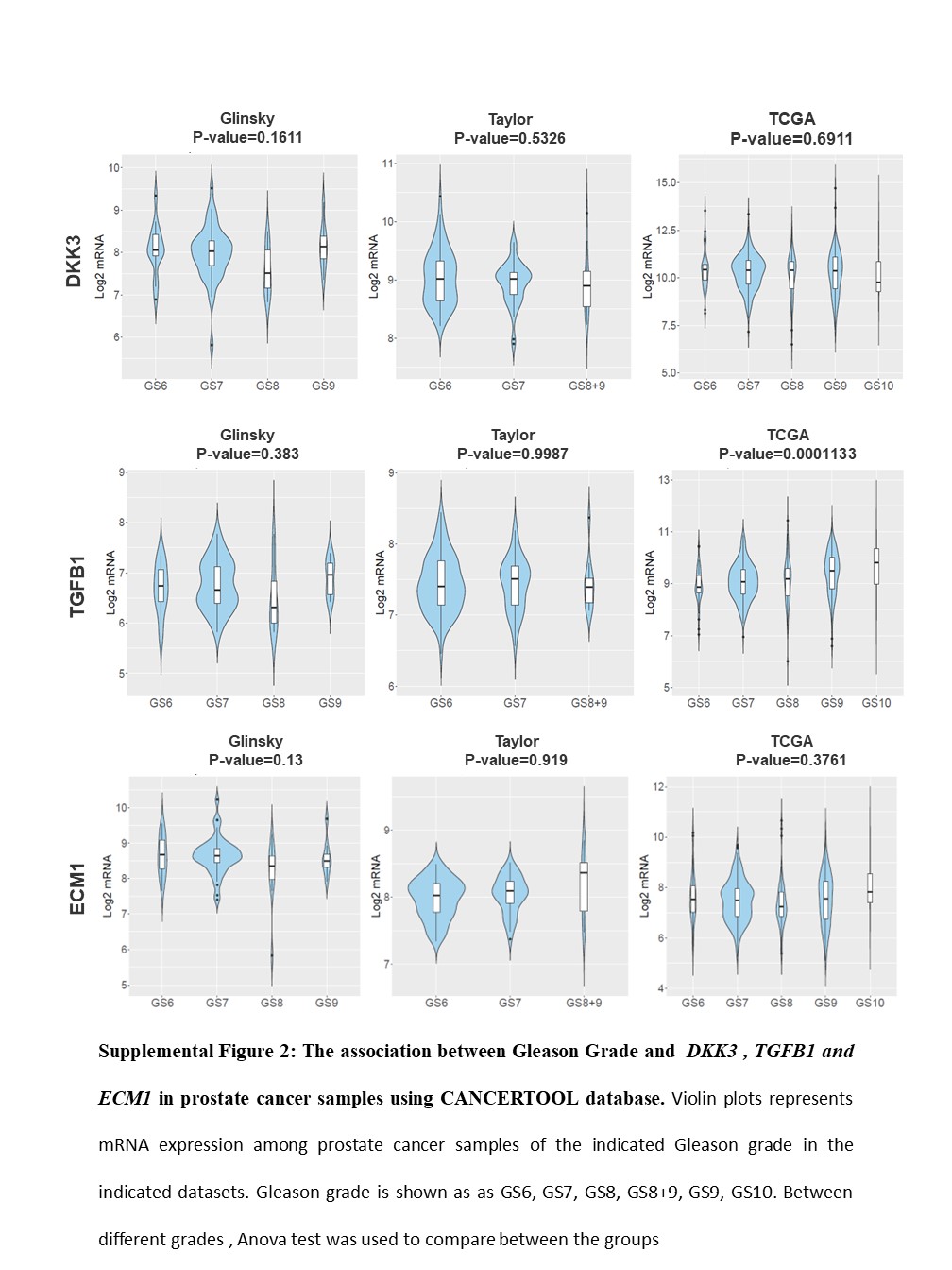

Supplement: Supplementary file 3 [file Image2.JPEG]
